# The complete mitochondrial genome and description of a new cryptic Brazilian species of *Metopiellus* Raffray (Coleoptera: Staphylinidae: Pselaphinae)

**DOI:** 10.7717/peerj.15697

**Published:** 2023-07-27

**Authors:** Angélico Asenjo, Marcus Paulo Alves de Oliveira, Renato R.M. Oliveira, Eder Soares Pires, Marcely Valois, Guilherme Oliveira, Santelmo Vasconcelos

**Affiliations:** 1 Instituto Tecnológico Vale, Belém, Pará, Brazil; 2 BioEspeleo Consultoria Ambiental, Lavras, Minas Gerais, Brazil; 3 Universidade Federal de Minas Gerais, Belo Horizonte, Minas Gerais, Brazil

**Keywords:** Amazon basin, Beetle, Brazil, Pselaphinae, Taxonomy

## Abstract

*Metopiellus* Raffray, 1908 is a genus of South American rove beetles typically found in tropical humid forests. Here we describe a new cryptic species from Eastern Amazon, in northern Brazil, *Metopiellus crypticus* Asenjo **sp. nov.**, and its major morphologic diagnostic features, which were photographed and illustrated. In addition, we bring the complete mitochondrial genome sequence of *M. crypticus* **sp. nov.**, and its position within the phylogenetic context of the family, including previously available mitogenomes of Staphylinidae species.

## Introduction

All species of the genus *Metopiellus* are distributed from Colombia to the North of Argentina (Asenjo et al., 2019; Fiorentino, Tocora & Ramirez, 2022). To date, species of the genus were recorded in the Colombian Amazon (*M. guanano* Fiorentino, Tocora & Ramirez, 2022), three species in the Brazilian Atlantic Forest (*M. aglenus* Reitter, 1885, *M. hirtus* Reitter, 1885, and *M. painensis* Asenjo, Ferreira & Zampaulo Rde, 2017), and one in Argentina (*M. sylvaticus* Bruch, 1933) (Asenjo et al., 2013; Asenjo, Ferreira & Zampaulo Rde, 2017; Fiorentino, Tocora & Ramirez, 2022). Members of *Metopiellus* are usually found inhabiting humid microenvironments on the forest floor consisting in decaying plant parts and their possible association with social insects as ants continue to be uncertain (Reitter, 1885; Park, 1942; Wasmann, 1894; Bruch, 1933). Asenjo, Ferreira & Zampaulo Rde (2017) found *M. painensis* inside the Loca dos Negros II and the Cerâmica caves in southeastern Brazil. This latter species was the only troglobitic Pselaphinae recorded from Brazil, up to date (Asenjo, Ferreira & Zampaulo Rde, 2017).

The aim of this study was to describe a new species from Brazil that was collected in the state of Pará, northern Brazil. The new species was found inhabiting forest areas, similar to other species in the genus, but it was also found in savanna-like environments. We described, for the first time, the complete mitochondrial genome of *Metopiellus crypticus* Asenjo **sp. nov.** positioning the new species in the phylogenetic context of Staphylinidae.

## Materials & Methods

### Field collection and sequencing

**Figure 1:**
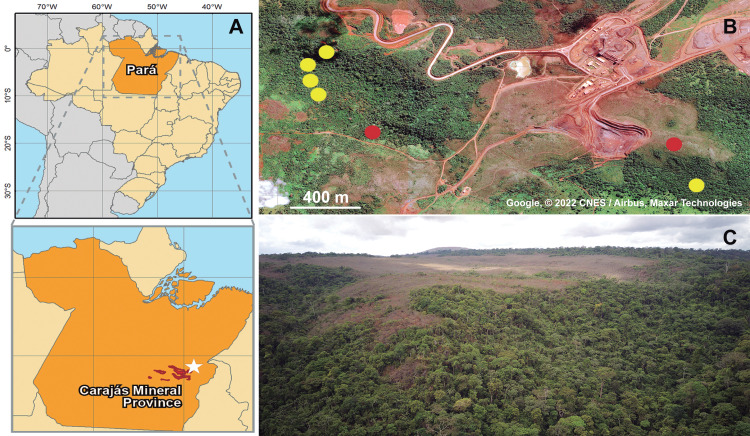
Geographic location of collected specimens of *Metopiellus crypticus* sp. nov. Brazil and Pará state are in orange, Carajás mineral Province in red and the specific type locality indicated by a white star (A); exact locations where the specimens were collected in the forest ground (yellow dots) and savanna regions (red dots) of Serra Leste (B) (Map data: Google, ©2022 CNES/Airbus, Maxar Technologies); panoramic view of the vegetation in Serra Leste, the savanna vegetations are in the flat area on mountain tops, and forest vegetation on the slopes and valley (picture by Alan Calux) (C).

A total of 12 specimens of *Metopiellus crypticus* Asenjo **sp. nov.** were collected in forest areas from the Serra dos Carajás in Pará, Brazil, in which the individuals were more abundant, and only two specimens from the *canga*, a savanna-like environment from the region (Figs. 1B–1C), according to the sampling permit 49.994, granted by ICMBio/MMA. Various collection methods were used in all sites (hand collection, litter sampling and hay-bait traps and soil sampling by flotation), but specimens were found only in litter-associated methods (hay-bait traps and soil sampling by flotation). Therefore, it is likely that the edaphic environment is characterized as a preferred habitat for populations of *Metopiellus crypticus* Asenjo **sp. nov.** The specimens were immediately fixed in 99% ethanol within a 2 mL centrifuge tube and transported to the laboratory.

Total genomic DNA was extracted from three specimens from the type population with the DNeasy Blood & Tissue kit (Qiagen), following the manufacturer’s protocol for insect samples, being deposited at the DNA bank of the Instituto Tecnológico Vale (ITV) under the accession numbers ITV10661, ITV21026 and ITV21027. Paired-end libraries were constructed from ∼50 ng of genomic DNA using the QXT SureSelect kit (Agilent Technologies, Santa Clara, CA, USA), with which the DNA samples were subjected to an enzymatic random fragmentation and simultaneously bound to adapters, following the manufacturer’s instructions. Then, the samples were purified and subjected to an amplification reaction using primers complementary to the adapters. Afterwards, the libraries were quantified using a Qubit 3.0 (Invitrogen, Waltham, MA, USA) fluorimeter and checked for fragment sizes in a 2100 Bioanalyzer (Agilent Technologies). Finally, the libraries were diluted in a solution of 0.1% Tris–HCl and Tween and pooled to be sequenced in an Illumina NextSeq 500 with the high-output v2 kit (300 cycles, 2 × 150 bp). Resulting raw sequencing reads with base quality <Phred 20 and length <70 bp were trimmed with AdapterRemoval v.2 (Schubert, Lindgreen & Orlando, 2016), resulting in 21,140,835 (ITV10661), 48,275,325 (ITV21026) and 49,851,478 (ITV21027) high quality pairs of reads, which were used to assemble the mitochondrial genomes using NovoPlasty 3.6 (Dierckxsens, Mardulyn & Smits, 2017). A bash script containing the commands for the cleaning and assembling steps is available as Data S1. Finally, the annotations were performed with MITOS2 (Bernt et al., 2013), with subsequent minor manual corrections using Geneious Prime 2021 (Biomatters), by comparing the annotated mitogenomes with previously available data for Pselaphinae species.

### Morphological study

*Specimens.* The apical segments were cleared in a double boiler using 10% KOH during three minutes. Dissections were made under a Leica S8APO (16×–128×) stereo-microscope. Pictures were obtained using the AxioCam 506 color camera connected to an Axio ZoomV16 (ZEISS) stereo microscope and Photoshop CC 2021 was used for image processing, with final plates being assembled in Adobe Illustrator CC 2021. Morphological character terminology, including foveation and its abbreviation followed Chandler (2001). All measurements were made using the Leica S8APO (16×–128×) stereo-microscope, and the width/length ratios were acquired using the widest and longest parts of the respective structures, being presented in millimeters, based on the holotype. We also performed additional measurements using the paratypes, comparing with the data obtained for the holotype (Data S2).

Measurements symbols:

BL body length (from margin of prolongation of head to tergite IX posterior margin)

BW body width (maximum width of elytra)

EL elytral length (maximum)

EW elytral width (maximum)

HL head length (from anterior margin of prolongation of head to head disc posterior margin)

HW head width (maximum)

NW neck width (minimum)

PL pronotum length (maximum)

PW pronotum width (maximum)

In the type label data, quotation marks (“ ”) separate different labels and a slash (/) separates different lines within a label. Text within square brackets [] is explanatory and is not included in the original labels.

Depositories. The specimens examined in this revision are deposited in the following collections (curators in parenthesis):

CEMT - Setor de Entomologia da Coleção Zoológica da Universidade Federal de Mato Grosso, Departamento de Biologia e Zoologia, Cuiabá, Mato Grosso, Brazil (Fernando Vaz-de-Mello).

ISLA - Coleção de Invertebrados Subterrâneos de Lavras, Setor de Zoologia, Departamento de Biologia, Universidade Federal de Lavras, Lavras, Minas Gerais, Brazil (Rodrigo Lopes Ferreira).

ITV - Coleção de DNA do Instituto Tecnológico Vale, Belém, Pará, Brazil (Santelmo Vasconcelos).

MPEG - Museu Paraense Emilio Goeldi, Belém, Pará, Brazil (Orlando Tobias Silveira).

### Nomenclatural acts

The electronic version of this article in Portable Document Format (PDF) will represent a published work according to the International Commission on Zoological Nomenclature (ICZN), and hence the new names contained in the electronic version are effectively published under that Code from the electronic edition alone. This published work and the nomenclatural acts it contains have been registered in ZooBank, the online registration system for the ICZN. The ZooBank LSIDs (Life Science Identifiers) can be resolved, and the associated information viewed through any standard web browser, by appending the LSID to the prefix http://zoobank.org/. The LSID for this publication is:

urn:lsid:zoobank.org:pub:85F589F2-AECD-4D52-B545-6363C4CE8E18

The online version of this work is archived and available from the following digital repositories: PeerJ, PubMed Central and CLOCKSS.

## Results

### Description

**Table:** 

Family Staphylinidae Latreille, 1802
Subfamily Pselaphinae Latreille, 1802
Tribe Metopiasini Raffray, 1904
Subtribe Metopiasina Raffray, 1904
Genus *Metopiellus*Raffray, 1908

### *Metopiellus crypticus* Asenjo, new species

**Figure 2:**
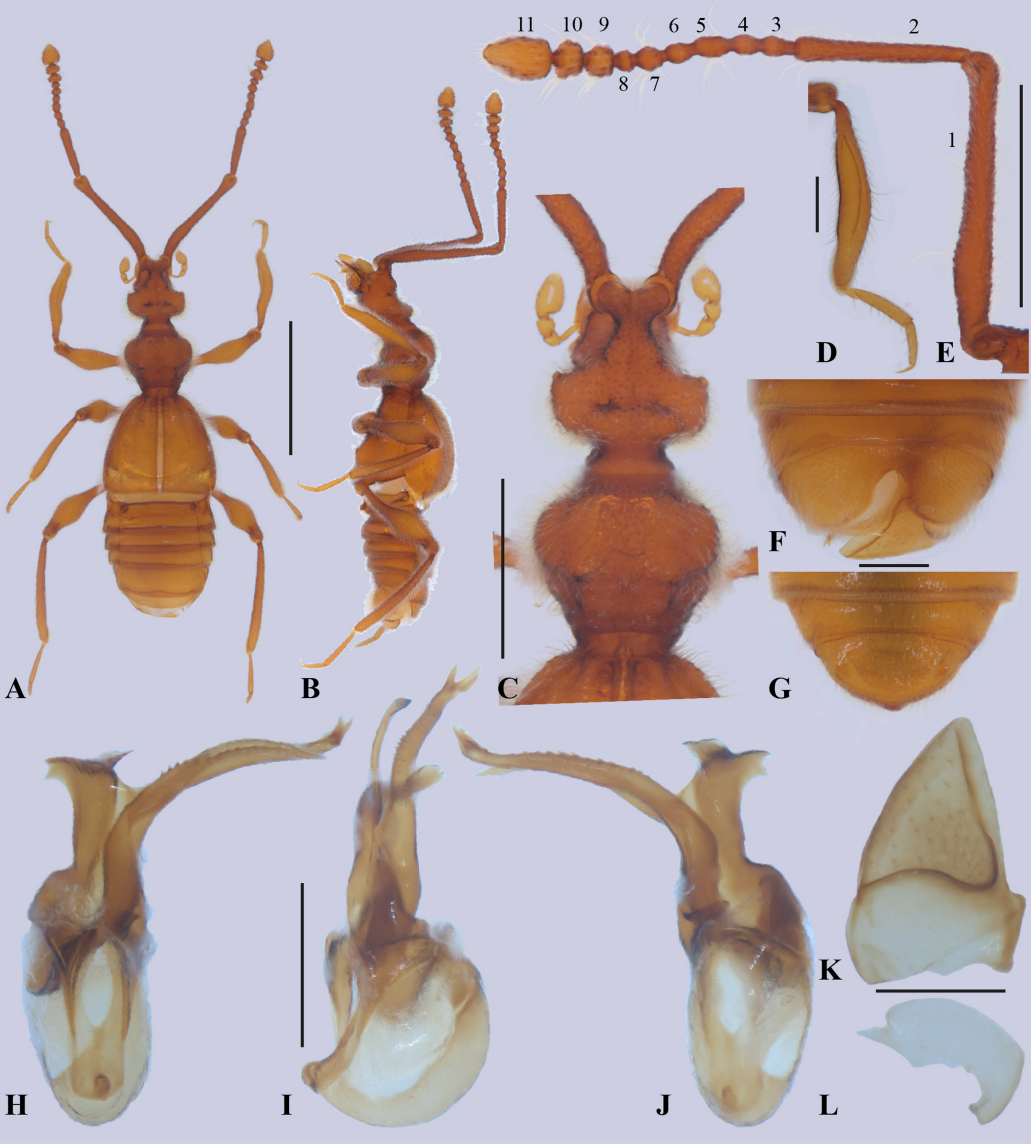
*Metopiellus crypticus* sp. nov. Habitus, dorsal view (A); habitus, left lateral view (B); head and pronotum, dorsal view (C); proleg (D); left antenna, lateral view (E); abdomen of male, ventral view (F); abdomen of female, ventral view (G); aedeagus, ventral view (H); aedeagus, lateral view (I); aedeagus, dorsal view (J); left tergum IX (K); right tergum IX (L). Scale bars: one mm (A–B); 0.5 mm (C, E); 0.2 mm (D, F–L). Holotype male (A–F, H–L). Paratype female (G).

**Figure 3:**
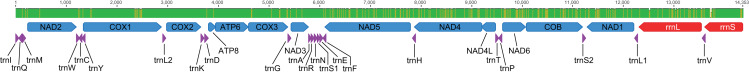
Representative genetic map of the mitogenome of *Metopiellus crypticus* sp. nov. Disposition of all 37 mitochondrial genes. Colored arrows pointing to the left and right represent the transcription regions of protein coding genes (blue), rRNA genes (red) and tRNA genes (purple) on the L and H strands, respectively. The green and brownish bars above the arrows indicate monomorphic and polymorphic nucleotide sites among the three analyzed genomes, respectively.

**Figure 4:**
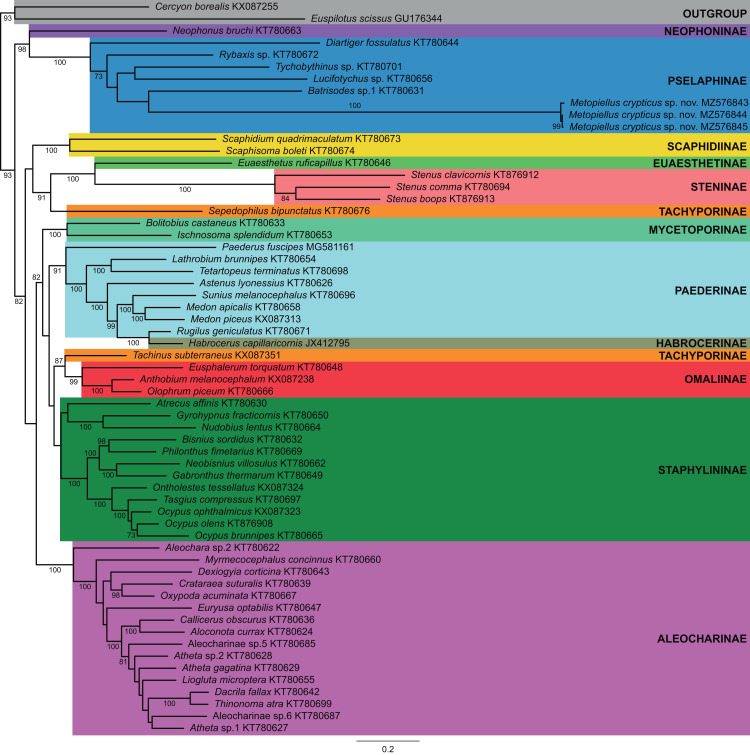
Mitogenome phylogenetic relationships among Staphylinidae species. Majority-rule consensus phylogram of the maximum likelihood analysis evidencing the phylogenetic relationships among Staphylinidae species with available mitogenomes in the GenBank database and the three specimens of *Metopiellus crypticus* **sp. nov.** sequenced here, indicating their respective subfamily affiliations. Well-supported groups (BS ≥ 70) are indicated by their bootstrap values near the branches.

(Figs. 1, 2, 3 and 4)

urn:lsid:zoobank.org:act:EE11C828-CCDD-4FDC-B090-A3CC80A1A2E1

*Type material* (seven males, two females). *Holotype:* BRAZIL, male, labeled “BRAZIL: Pará, /Curionópolis, Serra/Leste, 22M, 650137mE, /9339970mN, WGS84, [5°58′10.59″S, 49°38′36.86″W]/25.iv[April].2017, BioEspeleo *leg*”.; “Hay-bait trap, /Transect: T2, /Quadrant: E, Parcel: d”; “HOLOTYPE ♂ [red label]/*Metopiellus*/*crypticus* sp. nov./Desig. Asenjo et al. 2023” (CEMT-00120424). *Paratype:* (six males, two females), labeled: “BRAZIL: Pará, /Curionópolis, Serra/Leste, 22M, 652136mE, /9339073mN, WGS84, [5°8′39.63″S, 49°37′31.79″W]/26.iv[April].2017, BioEspeleo *leg*”.; “Soil sampling, /Transect: T3, /Quadrant: C, Parcel:-” (one male, ISLA-103823). “BRAZIL: Pará, /Curionópolis, Serra/Leste, 22M, 650360mE, /9339477mN, WGS84, [5°58′26.62″S, 49°38′29.57″W]/25.iv[April].2017, BioEspeleo *leg*”.; “Soil sampling, /Transect: T1, /Quadrant: D, Parcel:-” (one female, ISLA-103824). “BRAZIL: Pará, /Curionópolis, Serra/Leste, 22M, 652013mE, /9339211mN, WGS84, [5°58′35.15″S, 49°37′35.80″W]/26.iv[April].2017, BioEspeleo *leg*”.; “Soil sampling, /Transect: T4, /Quadrant: C, Parcel:-” (one male, MPEG-01051329). “BRAZIL: Pará, /Curionópolis, Serra/Leste, 22M, 650095mE, /9339732mN, WGS84, [5°58′18.34″S, 49°38′38.21″W]/25.iv[April].2017, BioEspeleo *leg*”.; “Hay-bait trap, /Transect: T2, /Quadrant: A, Parcel: d” (1 male, MPEG-01051330). “BRAZIL: Pará, /Curionópolis, Serra/Leste, 22M, 650095mE, /9339732mN, WGS84, [5°58′18.34″S, 49°38′38.21″W]/25.iv[April].2017, BioEspeleo *leg*”.; “Hay-bait trap, /Transect: T2, /Quadrant: A, Parcel: c” (two male, CEMT-00120425 and CEMT-00120426, and one female, CEMT-00120427). “BRAZIL: Pará, /Curionópolis, Serra/Leste, 22M, 650070mE, /9339845mN, WGS84, [5°58′14.67″S, 49°38′39.03″W]/25.iv[April].2017, BioEspeleo *leg*”.; “Hay-bait trap, /Transect: T2, /Quadrant: C, Parcel: b” (1 male, MPEG-01051331). All paratypes with label “PARATYPE [yellow label]/*Metopiellus*/*crypticus* sp. nov./Desig. Asenjo et al. 2023”.

*Additional specimens.* BRAZIL: Pará, Curionópolis, Serra Leste, 22M, 650137mE, 9339970mN, WGS84, [5°58′10.59″S, 49°38′36.86″W], 25.iv[April].2017, BioEspeleo leg., HBT-T2 E(B) (1 male, ITV10661). BRAZIL: Pará, Curionópolis, Serra Leste, 22M, 650070mE, 9339845mN, WGS84, [5°58′14.67″S, 4938′39.03″W], 25.iv[April].2017, BioEspeleo leg., HBT-T2 C(A), (1 female, ITV21026). BRAZIL: Pará, Curionópolis, Serra Leste, 22M, 650070mE, 9339845mN, WGS84, [5°58′14.67″S, 4938′39.03″W], 25.iv[April].2017, BioEspeleo leg., HBT-T2 C(A), (1 female, ITV21027).

*Diagnosis.* Among the members of *Metopiellus*, the new species *Metopiellus crypticus* Asenjo **sp. nov.** is very similar to *M. painensis* because both have a similar habitus (Figs. 2A–2B; Fig. 1 in Asenjo, Ferreira & Zampaulo Rde, 2017) and eyes nearly absent (Figs. 2A–2C; Fig. 3 in Asenjo, Ferreira & Zampaulo Rde, 2017), but the new species differs by having the antennomere 7 rounded (Fig. 2E) (rectangular in *Metopiellus painensis* [Fig. 5 in Asenjo, Ferreira & Zampaulo Rde, 2017]). Furthermore, *Metopiellus crypticus* Asenjo **sp. nov.** further differs by the paramere asymmetric elongate with apex bifurcated (Figs. 2H–2J) (paramere asymmetric not bifurcated in *M. painensis* [Figs. 10–12 in Asenjo, Ferreira & Zampaulo Rde, 2017]). Also, *Metopiellus crypticus* **sp. nov.** differs by the median lobe curved and edge with long line of small teeth (Figs. 2H–2J) (median lobe almost right with the apex curved without small teeth in *M. painensis* [Figs. 10–12 in Asenjo, Ferreira & Zampaulo Rde, 2017]).

Holotype male, BL: 2.68. Body, mouthparts, antennae and tarsi light brown (Figs. 2A–2B).

Head: pyriform (Figs. 2A and 2C), length (HL: 0.44) similar to width (HW: 0.44), anterior region narrower and ending in an emarginated, antennal tubercle. Posterior margin of head narrowing, with posterolateral angles rounded. Neck almost half of width (NW: 0.19) of head. Head with two vertexal foveae [VF] (Figs. 2A and 2C), foveae connected by a transverse sulcus near posterior margin. Vertex longitudinally impressed, with weak sulcus running from anterior margin of antennal tubercle to neck. Ventral surface of head without gular sulcus and posterior region with two gular foveae [GF] connected by curved sulcus. Eyes (Figs. 2A–2C) composed of some ommatidia situated at middle of head length in lateral view. Antennae (Fig. 2E) almost 3/4 body length, scape almost half antenna length, last three antennomeres gradually broadening. Scape length 0.92 mm, width 0.09 mm, pedicel shorter than scape (0.39: 0.08), antennomere 3 (0.06: 0.06), antennomere 4 (0.05: 0.06), antennomere 5 (0.07: 0.06), antennomere 6 (0.06: 0.06), antennomere 7 (0.06: 0.08), antennomere 8 (0.03: 0.06), antennomere 9 (0.06: 0.1), antennomere 10 (0.06: 0.12), antennomere 11 (0.15: 0.14); all antennomeres covered by long microsetae.

Thorax: pronotum (Figs. 2A, 2C) slightly wider than long (PL: 0.45; PW: 0.52) widest at anterior half. Pronotum convex with weak median longitudinal sulcus, each side with lateral sulcus, with transversal antebasal sulcus. Pronotum with basal and anterior margins weakly emarginated; with median antebasal fovea (MAF) and lateral antebasal fovea (LAF). Prosternum with lateral procoxal fovea (LPCF). Mesoventrite with median mesocoxal fovea (MMNF), lateral mesosternal foveae (LMNF) lateral mesocoxal foveae (LMCF), and with lateral metasternal foveae (LMTF). Metaventrite with median metasternal fovea (MMTF) and one flat median triangle area before metacoxal cavities.

Elytra: subquadrate (EL: 0.74; EW: 0.80), sides gradually broadening apically (Fig. 2A). Posterior margins slightly concave, discal stria (DS) and sutural stria (SS) present. Elytron with two basal elytral foveae (BEF) at anterior margin, one at side of base of the elytral sutural stria, second on the base of discal stria. Apico-lateral margin of elytra with a small notch. Flight wings absent.

Legs: Legs long and slender (Figs. 2A–2B). Femora thickened in apical half. Tibiae curved and similar in length to femora, all tibiae thickened at apex. Protibiae carinate and open at base, lacking microsetae on the concave mesial face (Fig. 2D). Tarsi 3-segmented (Fig. 2D), first tarsomeres short, last 2 tarsomeres longer, tarsomere 2 longer than segment 3; all tarsi with single claw and minute accessory seta. Procoxae conical and prominent, mesocoxae rounded and prominent, metacoxae transverse, region articulated with trochanter conical in shape. Procoxae with small, apically pointed prosternal process, mesocoxae weakly separated, metacoxae contiguous.

Abdomen: strongly margined (Fig. 2A), with five visible tergites (morphological tergites IV-VIII), tergite III reduced to translucent plate beneath elytra, tergite IV with basolateral fovea (BLF), tergite VIII with apex straight. Tergites IV-VII bordered by distinct paratergites, paratergite in abdominal segment IV with one small tooth in the middle. Sternite III with transverse depressed plate completely bare and beneath metacoxae, transverse plate with longitudinally projecting carina at middle. Sternite IV with baselateral fovea (BLF). Tergum IX divided into two plates; right plate (Fig. 2K) larger and more sclerotized than left (Fig. 2J). Sternite VIII (Fig. 2F) with apex deeply emarginate.

Aedeagus (Figs. 2H–2J): asymmetric with paramere partially fused to form elongate plate with apex forked, the median lobe slightly bulbous at base, elongate and narrow, stronger curved laterally at apex, on edge with line of small teeth.

*Female*. Similar to male, except apex of tergite VIII convex (Fig. 2G).

Distribution. Only known from Serra Leste, Curionópolis, Pará, Brazil (Figs. 1A–1C).

Etymology. The specific epithet “crypticus” is a noun in apposition.

### Mitogenome sequence and phylogenetic placement

All three assembled mitogenomes (GenBank accession numbers MZ576843 (ITV10661), MZ576844 (ITV21026) and MZ576845 (ITV21027)) presented the standard structure sequence and gene content for Metazoa, consisting of 13 PCG, 22 tRNA genes and two rRNA genes (Fig. 3). The three mitogenome assemblies ranged in size from 14,353 to 14,984 bp, with similar GC contents between 16.2% and 16.5%, and 98.3% identical sites (1.7% differences). We observed differences in nucleotide composition among the three mitogenomes of *Metopiellus crypticus* Asenjo **sp. nov.**, with indel events mostly occurring in the rRNA genes (three in each locus). Also, rrnL presented 33 site substitutions as indicated by the mismatches in the alignment, one of the highest proportions of polymorphic sites within the analyzed mitogenomes (2.68% of the 1232 bp), behind only of NAD6 (3.73% of the 456 bp), excluding the tRNAs, which are considerably shorter with 63 bp on average (Table 1).

**Table 1:** General features of the mitochondrial genes of *Metopiellus crypticus* sp. nov.

**Gene**	**Size (bp)**	**Indels**	**Mismatches**	**% Mismatches**	**Coding strand**	**Start codon**	**Stop codon**
ATP6	651	0	0	0.00	L	ATG	TAA
ATP8	153	0	1	0.65	L	ATT	TAA
COB	1110	0	26	2.34	L	ATA	TAA
COX1	1537	0	11	0.72	L	ATT	T
COX2	682	0	0	0.00	L	ATA	T
COX3	781	0	8	1.02	L	ATG	T
NAD1	924^a^	1	18	1.95	H	ATA	TAA
NAD2	963	0	1	0.10	L	ATA	TAA
NAD3	348	0	2	0.57	L	ATT	TAG
NAD4	1332^a^	0	29	2.18	H	ATG	TAA
NAD4L	273	0	4	1.47	H	ATT	TAA
NAD5	1692	0	35	2.07	H	ATT	TAA
NAD6	456^a^	1	17	3.73	L	ATT	TAA
rrnL	1232	3	33	2.68	H	–	–
rrnS	728^a^	3	4	0.55	H	–	–
trnA (tgc)	52	0	0	0.00	L	–	–
trnC (gca)	63^a^	1	0	0.00	H	–	–
trnD (gtc)	64	0	1	1.56	L	–	–
trnE (ttc)	62	0	0	0.00	L	–	–
trnF (gaa)	63^a^	1	1	1.59	H	–	–
trnG (tcc)	63	0	2	3.17	L	–	–
trnH (gtg)	63	0	1	1.59	H	–	–
trnI (gat)	63	0	0	0.00	L	–	–
trnK (ctt)	68	0	0	0.00	L	–	–
trnL1 (tag)	61	0	0	0.00	H	–	–
trnL2 (taa)	62	0	0	0.00	L	–	–
trnM (cat)	68^b^	1	0	0.00	L	–	–
trnN (gtt)	63	0	0	0.00	L	–	–
trnP (tgg)	64^a^	1	0	0.00	H	–	–
trnQ (ttg)	63	0	0	0.00	H	–	–
trnR (tcg)	60	0	0	0.00	L	–	–
trnS1 (tct)	55	0	0	0.00	L	–	–
trnS2 (tga)	64	0	2	3.13	L	–	–
trnT (tgt)	64	0	0	0.00	L	–	–
trnV (tac)	64	0	2	3.13	H	–	–
trnW (tca)	64^a^	1	0	0.00	L	–	–
trnY (gta)	63	0	1	1.59	H	–	–

**Notes.**

Sequenced mitogenomes based on from three different specimens, indicating the size of the transcription regions, presence of indel events, number of mismatches after the alignment of the mitogenomes, coding strand, and sequences of both start and stop codons.

a For genes with indel events, we presented the length observed in two of the three specimens.

b For trnM (cat), the value presented correspond to the average size among the three mitogenomes.

Most genes were encoded in the L-strand, including nine PCGs (ATP6, APT8, COB, COX1, COX2, COX3, NAD2, NAD3 and NAD6) and 14 tRNA genes (trnA, trnD, trnE, trnG, trnI, trnK, trnL2, trnM, trnN, trnR, trnS1, trnS2, trnT and trnW). Also, ATT was the most frequent start codon, being observed in seven genes, followed by ATA in four genes, and ATG in three (Table 1). On the other hand, almost all genes presented the TAA stop codon, except for NAD3 with TAG, and the three COX genes with an incomplete stop codon (Table 1).

Previously published mitogenome sequences of Staphylinidae species from 11 subfamilies, plus one species of Hydrophilidae (*Cercyon borealis*) and one of Histeridae (*Euspilotus scissus*) to be used as outgroups, were obtained from GenBank, totaling 61 accessions. Sequences of the 13 protein coding genes (PCG) were aligned with MAFFT v7.45 (Katoh et al., 2002; Data S3) and maximum likelihood (ML) phylogenetic trees were obtained using RAxML v8 (Stamatakis, 2014), implemented in raxmlGUI v2 (Edler et al., 2021) using the model GTR+PROTGAMMA and the rapid bootstrapping option with 1,000 replicates (Data S4).

In the phylogenetic analysis, most of the subfamilies were recovered as monophyletic and well supported, except for Tachyporinae and Paederinae, which were polyphyletic and paraphyletic, respectively, and Staphylininae, presenting a low bootstrap support (BS = 55) (Fig. 4). Within Pselaphinae, the relationships among the sampled species were mostly unsupported (BS <70). The three specimens of *Metopiellus crypticus* **sp. nov.** grouped with maximum statistical support (BS = 100) in the longer branch within the subfamily, being recovered as sister to *Batrisodes* sp., although with low statistical support (BS = 42) (Fig. 4).

## Discussion

The new species belongs to the genus *Metopiellus* based on the third antennal segment being much shorter than the second (Fig. 2E); the posterior coxae contiguous or nearly so; and the mesial face of protibia being carinate and open at its base and apex (Fig. 2B) (Raffray, 1908; Park, 1942; Asenjo, Ferreira & Zampaulo Rde, 2017). One of the characters “pronotum not being spinose” for *Metopiellus*, should not be considered a good character as considerated by previous authors to define the genus since *M. guanano* has pronotum with four small spines (Fiorentino, Tocora & Ramirez, 2022).

Specimens of the known species on the genus *Metopiellus* were collected in litter of ants or in caves (Asenjo, Ferreira & Zampaulo Rde, 2017). Unlike the other species of the genus, which have been recorded in forested areas or inside caves, the new species has been found in forested areas of the Serra dos Carajás, as well as in a savanna-like environment, although being less abundant in the latter.

For the first time, the mitochondrial genome of a *Metopiellus* species is described focusing on the phylogenetic position of *Metopiellus crypticus* **sp. nov.** within the subfamily Pselaphinae. In the obtained topology, the new species was recovered as sister to *Batrisodes* sp., although with low statistical support (BS = 42). However, this grouping was probably an artefact influenced by the absence of published mitogenomes of the others representatives of the other Metopiasini.

Despite of all three assembled mitogenomes presenting the standard structure sequence and gene content for Metazoa, we could not obtain a circularized assembly for any of them, probably due to a high repetitive DNA content in the D-loop control region (Sayadi et al., 2017), as indicated by the several mononucleotide repeats in both ends of the assembled sequences. Such a pattern has been frequently reported for beetle species, with several Coleoptera mitogenomes available in the GenBank database containing all expected genes, but missing part of the control region, and thus being reported as partial sequences.

##  Supplemental Information

10.7717/peerj.15697/supp-1 Supplemental Information 1 Mitogenome of Metopiellus crypticus, accession no. ITV10661, GenBank no. MZ576843

10.7717/peerj.15697/supp-2 Supplemental Information 2 Mitogenome of Metopiellus crypticus, accession no. ITV21026, GenBank no. MZ576844

10.7717/peerj.15697/supp-3 Supplemental Information 3 Mitogenome of Metopiellus crypticus, accession no. ITV21027, GenBank no. MZ576845

10.7717/peerj.15697/supp-4 Data S1 Bash script for cleaning Illumina reads and assembling mitogenomes

10.7717/peerj.15697/supp-5 Data S2 Metopiellus crypticus sp. nov. measurements, comparing the holotype specimen with eight paratypes

10.7717/peerj.15697/supp-6 Data S3 Amino acid sequence matrix of Staphylinidae species using the 13 protein coding genes of the sampled mitogenomes

10.7717/peerj.15697/supp-7 Data S4 Commands used to run the maximum likelihood analysis in RAxML

## Additional Information and Declarations

### Field Study Permissions

The following information was supplied relating to field study approvals (*i.e.*, approving body and any reference numbers): The samples were collected under the sampling permit 49.994, as granted by ICMBio/MMA.

### DNA Deposition

The following information was supplied regarding the deposition of DNA sequences: The assembled mitogenomes are available at GenBank: MZ576843, MZ576844 and MZ576845.

### Data Availability

The following information was supplied regarding data availability: The troglogen data is available at NCBI: PRJNA862473.

### New Species Registration

The following information was supplied regarding the registration of a newly described species: Publication LSID: urn:lsid:zoobank.org:pub:85F589F2-AECD-4D52-B545-6363C4CE8E18 *Metopiellus crypticus*: urn:lsid:zoobank.org:act:EE11C828-CCDD-4FDC-B090-A3CC80A1A2E1
